# Durability of Epoxy Adhesives and Carbon Fibre Reinforced Polymer Laminates Used in Strengthening Systems: Accelerated Ageing versus Natural Ageing

**DOI:** 10.3390/ma14061533

**Published:** 2021-03-21

**Authors:** Ricardo Cruz, Luís Correia, Aloys Dushimimana, Susana Cabral-Fonseca, José Sena-Cruz

**Affiliations:** 1 Institute for Sustainability and Innovation in Structural Engineering (ISISE)/Institute of Science and Innovation for Bio-Sustainability (IB-S), University of Minho, Azurém, 4800-058 Guimarães, Portugal; a51314@alumni.uminho.pt (R.C.); lcorreia@civil.uminho.pt (L.C.); aloysdushimimana@yahoo.fr (A.D.); 2 Laboratório Nacional de Engenharia Civil, Materials Department, Av. do Brasil 101, 1700-066 Lisboa, Portugal; sbravo@lnec.pt

**Keywords:** epoxy adhesive, CFRP laminate, durability, natural outdoor ageing, artificial accelerated ageing

## Abstract

This work addresses the durability of structural epoxy adhesives and carbon fibre reinforced polymer (CFRP) laminates typically used in strengthening of existing reinforced concrete structures exposed to natural ageing. The experimental program included four natural (real) outdoor environments inducing ageing mainly caused by carbonation, freeze-thaw attack, elevated temperatures, and airborne chlorides from seawater. Moreover, a control (reference) environment (20 °C of temperature and 55% of relative humidity) and an environment involving water immersion of the materials under controlled temperature (20 °C of temperature) were also included in this investigation. The characterization involved the assessment of the physical, chemical and mechanical properties along a study period of up to two years. Furthermore, comparisons between the natural ageing tests developed in the scope of the present work and accelerated ageing tests existing in the literature were performed. Regarding to the epoxy adhesives, an increase in the glass transition temperature with the time was observed, while the tensile properties decreased, regardless of the outdoor environment. The CFRP laminates were marginally affected by the studied environments. Despite the remarkable dispersion of the results observed in the accelerated ageing tests for the period investigated, this testing protocol yielded higher mechanical degradation than under natural ageing.

## 1. Introduction

Structural repairing and strengthening of existing reinforced concrete (RC) structures with systems involving fibre reinforced polymer (FRP) materials are considered state-of-the-art in civil engineering. Carbon FRP (CFRP) materials are typically used due to, mainly, their superior mechanical properties (higher stiffness, strength and fatigue life, among others), and their great resistance to aggressive environments [1,2,3,4,5,6,7].

CFRP materials are commonly applied using the externally bonded reinforcement (EBR) technique or the near surface mounted (NSM) strengthening technique. The EBR technique uses laminate strips or sheets which are externally bonded to the surface of the structural member to be strengthened, while in the NSM technique the reinforcements (laminates or bars) are inserted into grooves cut into the concrete cover of the structural member. Typically, epoxy adhesives are used as the bonding agent. Both techniques are suitable for flexural and shear strengthening. In some cases, active techniques, e.g., prestressing, are required. The use of prestressing combines the advantages of FRP systems with external prestressing, which leads to more efficient use of concrete and CFRP and reduction on the deflection and crack width, amongst other advantages [8,9].

The knowledge on the durability of RC structures strengthened with CFRP is essential for structural safety. The durability has been intensively studied under laboratory conditions using accelerated ageing protocols. These types of protocol typically use higher stress levels, extreme environmental actions and/or size adjustments (i.e., a reduction of the sample thickness) in order to accelerate the degradation process and reduce the experimental time [10,11]. Then, the results are extrapolated to the real outdoor conditions and/or real-scaled elements. However, a very few studies on the durability have been performed under real outdoor conditions (natural ageing). Moreover, the relationship between accelerated ageing tests performed under laboratory conditions and natural ageing conditions for assessing the durability of these systems is not fully understood [12].

Typically, outdoor environments offer a combination of several degradation agents, such us moisture, temperature, and ultraviolet (UV) radiation. The absorption of moisture on composite materials can result from their exposure to precipitation, humidity or aqueous solutions diffused through other substrates. The degradation effect of moisture on composite materials can be understood by considering its effect on the constituent elements of the system, which are the fibre, the matrix and the fibre-matrix interphase. The absorption of moisture mainly damages the resin, which may lead to changes in the structure of the polymer [13]. Nevertheless, moisture can also deteriorate the interphase fibre-matrix, by reducing the fibre-matrix bond, and lead to the degradation at the fibre level. The diffusion of water in polymers and adhesives may lead to changes in their mechanical, physical, and chemical properties [14]. Physical ageing consists in a reversible change of material properties which can be recovered, in part, after drying (the property changes are also dependent on the temperature). Physical ageing includes a mechanism commonly known as plasticization, which results in the reduction in the modulus of elasticity and strength, and an increase in the ductility [15,16]. Another physical mechanism is swelling, which consists of volumetric changes as a result of the moisture content alone, independently of thermal expansion. Potentially, swelling can affect the fibre-matrix interface bond, leading to premature cracking or fibre separation [17]. The presence of water can also lead to a reduction of the glass transition temperature (*T*_g_), that identifies the interval of temperature above which the mechanical properties (stiffness and strength) of the epoxy adhesive or polymer matrix drop drastically [3,16,17].

Regarding chemical ageing, it can occur after longer exposure to moisture. This is mostly an irreversible process of degradation which occurs in all constituent elements of the system (fibre, matrix and interface fibre-matrix) [13]. Finally, the mechanical degradation may result from the combination of different chemical and physical mechanisms, as swelling leads to microcracking in the weakened resin after hydrolysis as well as debonding effects at the interface [18]. The degradation level caused by moisture is highly influenced by the type of fibre reinforcement, and CFRP materials are relatively immune to the effects of moisture [3,10,16,17]. In fact, a recent study on the durability of CFRP laminate strips [10], reported a 3% reduction on the tensile strength and elastic modulus after 240-day exposure to full immersion in tap water. However, the synergy between moisture and other degradation agents can have a combined effect on CFRP laminates. In fact, Cabral-Fonseca et al. [19] investigated the synergistic effect of water-based environments, including immersion in: (i) demineralized water, (ii) water with 35 g/L of sodium chloride, and (iii) in an alkaline solution and different temperatures (60 °C, 40 °C and 23 °C), on the durability of three CFRP laminates typically used for construction applications. A reduction on the flexural strength was observed for all three environments. The results clearly showed higher rates of degradation at the more elevated temperature (60 °C), between 32% and 11% for the demineralized water. In contrast, epoxy adhesives are highly susceptible to moisture effects. Silva [10] carried out ageing tests on epoxy adhesive and observed relevant reductions of 14%, 47% and 38%, respectively, in the *T*_g_, elastic modulus and tensile strength of the adhesive, after being fully immersed in water for a period of 480 days. In another investigation on the durability of a commercial epoxy adhesive, Sousa et al. [20] reported high reductions on the flexural properties (strength: −24%; and elastic modulus: −30%) after immersion in water at 40 °C for two years.

The thermal effects on composites may be due to: (i) sub-zero temperatures, (ii) freeze-thaw cycles, (iii) thermal cycles, and/or (iv) elevated temperatures. Relevant literature review works about this topic can be found in [16,21,22,23,24,25]. The exposure to elevated temperatures leads, mainly, to softening of the resin (viscous response) and therefore, of the composite material. The stiffness and strength of resins and composite material are dependent of the temperature, and elevated temperatures near the glass transition temperature lead to softening and an increase in the viscoelasticity of the polymeric matrix of a FRP material or the adhesive. In contrast, the presence of elevated temperatures may lead to the positive post-cure phenomenon. As referred in [3], high temperatures can act as a post-cure of the material and thus increase *T*_g_. In fact, an experimental work carried out by Cromwell et al. [26] showed an improvement on the tensile properties (strength and elastic modulus) of CFRP strips after exposure to a dry heat (60 °C) environment for periods of 6 and 18 weeks. An increase in moisture absorption and diffusion can also occur in the presence of elevated temperatures. Furthermore, the synergy between moisture and temperature might lead to higher degradation effects than each single individual environmental agent [16,21]. In general, thermal cycles lead to small changes on the stiffness and strength of FRP materials, unless the temperature variation is extremely high. On the other hand, in composite materials with high modulus resins, thermal cycles may lead to the appearance of microfractures. Regarding freeze-thaw cycles, the performance of reinforcing fibres, in general, is not affected. However, the performance of the resin and of the fibre-resin interface are reduced when FRP materials are exposed to freeze-thaw cycles, as a result of the difference in the coefficients of thermal expansion (CTE) between the polymer matrix and reinforcing fibres. Sub-zero temperatures can lead to the development of higher strength and stiffness values which can enhance the performance of polymer resin-based systems, however, freeze-thaw cycles (incursions to sub-zero temperatures) in the presence of moisture, can lead to increases in the degradation of the properties of the FRP materials. At their service temperatures, CFRPs are normally immune to thermal cycles [27]. However, since carbon fibers and the matrix resin present different CTEs, this might lead to matrix microcracking and might increase the degradation ratio. Silva [10] studied the effect of: (i) freeze-thaw cycles (temperature range: −18 °C to 20 °C; duration: 240 days) and (ii) thermal cycles (temperature range: 20 °C to 80 °C/−15 °C to 60 °C; duration: 240 days/180 days) on CFRP laminate strips, and reported negligible variations on the tensile strength, ultimate strain or stiffness of the laminate. However, the effect of the same environments on a cold-curing epoxy adhesive led to a significant reduction on the *T*_g_ of the epoxy adhesive (of 23%), after the freeze-thaw ageing, as the curing process was interrupted due to the low temperatures, and an increase on the tensile properties (strength: 18% to 50%; elastic modulus: 5% to 25%), after the thermal cycles, due to post-curing phenomenon [10]. It should be referred that temperature is a key factor in the curing of the epoxy adhesive. Moussa et al. [28] studied the influence of low temperatures on the curing process of a cold-curing epoxy adhesive and observed a considerable increase in the curing time when lower temperatures were considered: for high temperatures (60 °C to 35 °C), full curing was attained after a few hours (3.7 to 1.6 h), whereas a low temperature (10 °C) led to longer curing periods (3 days).

Ultraviolet (UV) radiation can affect FRP structures used in outdoor applications. The influence of UV radiation on composite materials has been reported in several literature reviews [29,30,31]. FRP material damage by UV radiation mainly affects the components of the polymer matrix. As one of its functions is the transferring of stresses to and between the reinforcing fibres, the degradation caused in these component may strongly affect the mechanical properties of the composite material [29]. As demonstrated by some investigations on this topic [16], the effects of exposure to UV radiation, also known as photodegradation, on composite materials are mainly located in the top few microns of the surface, and affects mainly the aesthetic properties (loss of gloss and discolouration, often referred to as yellowing). It has been shown that, in some cases, the damage to the material surface disproportionally affects the thermomechanical properties of FRP composites, leading to flaws and to fracture initiation at reduced stress levels when compared with those measured on unexposed material. It should be also mentioned that such flaws can lead to the ingress of moisture. Indeed, for outdoor environments, the effect of UV is often combined with the action of temperature, moisture, wind-borne abrasives, freeze-thaw cycling, and other environmental factors [16]. According to [3], carbon fibres are practically unaffected by UV radiation and, in general, the mechanical properties of composites are only slightly influenced by UV exposure. In an experimental study on the durability of three commercial CFRP laminate strips, Cabral-Fonseca et al. [19] observed photodegradation on the surface of the CFRP; however, the laminate did not show any variations on the flexural strength after 2000 h exposure to ultraviolet radiation. Sousa et al. [20] studied the effect of UV radiation, alongside with moisture and temperature cycles in an outdoor environment (Mediterranean climate), on commercial epoxy adhesives. The results showed inconsistent variations of the adhesive properties (shear modulus: +13%; shear strength: −7%; glass transition temperature: +2% reduction). A post-curing process during the outdoor ageing was reported as the cause that led to the increase in the shear strength. The effect of laboratory and outdoor environments on the flexural properties and curing of two epoxy adhesives was investigated by Frigione et al. [32,33]. The outdoor environment (in Salerno, Italy), which included UV radiation, along with temperature and humidity fluctuations, led to negligible variations of the mechanical properties of the adhesives.

Despite the significant number of studies that have been performed concerning the durability topic, gaps in the existing knowledge can be found, mainly concerning to the performance of this type of materials under natural ageing. Another important issue that needs to be addressed is the relationship between the effects of ageing under laboratory conditions (accelerated ageing) and outdoor conditions (natural ageing). This paper presents the results of an investigation on the durability of epoxy adhesives and CFRP laminates subjected to four outdoor environments inducing ageing mainly by exposure to carbonation, freeze-thaw attack, elevated temperatures, and airborne chlorides from seawater for up to two years. A control (reference) environment and an environment involving water immersion of the materials under controlled temperature were also included. Along the time of the experiments, the physical, chemical and mechanical properties of the materials were characterized, at certain timepoints, namely, after production (T0), and after one (T1) and two (T2) years of exposure. Furthermore, the results of natural ageing test developed in this work and accelerated ageing tests described in the literature were compared.

## 2. Materials, Experimental Program and Methods

### 2.1. Materials

#### 2.1.1. Epoxy Adhesives

This research work included the study of two commercial cold curing epoxy adhesives: (i) S&P Resin 220 epoxy adhesive, supplied by S&P^®^ Clever Reinforcement Ibérica Lda. Company (Seixal, Portugal, hereupon referred as ADH1 adhesive; and, (ii) the trademarked Sikadur-30 epoxy adhesive, supplied by SIKA Schweiz AG (Zurich, Switzerland), henceforth referred to as ADH2 adhesive. Both epoxy adhesives are typically used in the context of strengthening concrete structures with CFRP laminates. Table 1 presents the characteristics declared by each supplier.

#### 2.1.2. CFRP Laminates

The CFRP laminate strips used in this research work are produced by S&P^®^ Clever Reinforcement Ibérica Lda. Company and trademarked as CFK 150/2000. Prefabricated by pultrusion, these CFRP laminates are composed of unidirectional carbon fibres (fibre content higher than 68%) held together by a vinyl ester resin matrix. Two distinct geometries were analysed: (i) the laminate with the cross section of 10 mm by 1.4 mm, later referred as L10 strip, typically used with the NSM strengthening technique and (ii) the laminate with the cross section of 50 mm by 1.2 mm, hereupon referred to as L50 strip, normally used for external bonded applications (EBR). Both CFRP strips present a black and smooth external surface. According to the supplier, the mean value of the elastic modulus is higher than 170 GPa and characteristic tensile strength is higher than 2000 MPa [44].

### 2.2. Experimental Program

**Figure 1:**
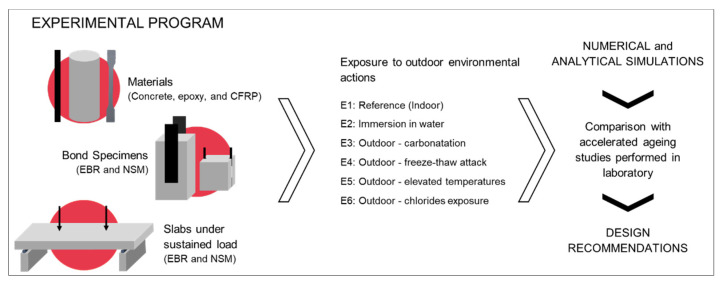
Flowchart of the FRPLongDur Project.

The experimental program was carried out in the framework of the “FRPlongDur—Long-term structural and durability performances of reinforced concrete elements strengthened in flexure with CFRP laminates” project. The main objective of the FRPLongDur project is to contribute to the knowledge on the long-term structural behaviour and durability performance of reinforced concrete RC elements strengthened in flexure with CFRP laminates under relevant artificial and real environmental conditions. Figure 1 shows the flowchart of the research project, which includes full-scale RC slabs strengthened in flexure with CFRP laminates throughout the NSM and EBR techniques, bond specimens and samples of the involved materials (concrete, epoxy adhesive and CFRP laminate). The present work addresses only the epoxy adhesives and the CFRP laminates.

Six different environmental conditions were studied (see also Table 2): two artificial environments (E1 and E2) and four outdoor environments (E3 to E6). The environment E1 was considered as the reference (controlled hygrothermal conditions, 20 °C/50% RH), while environment E2 intends to understand the effect water immersion under controlled temperature (approximately 20 °C). Despite the fact that all the remaining outdoor (natural) environmental conditions were located in Portugal, given the selected places (herein named experimental stations) it is expected to achieve specific ageing conditions, namely: E3—higher levels of concrete carbonation (due to the levels of CO_2_ concentration since the experimental station is placed near the International Airport of Lisbon and near a critical highway); E4—freeze-thaw cycles, since the experimental station is located close to the highest mountain of Portugal (‘Serra da Estrela’); E5—higher (elevated) service temperatures and lower relative humidity; E6—higher levels of chlorides concentration and relative humidity, since the station is placed near the Atlantic Ocean.

**Figure 2:**
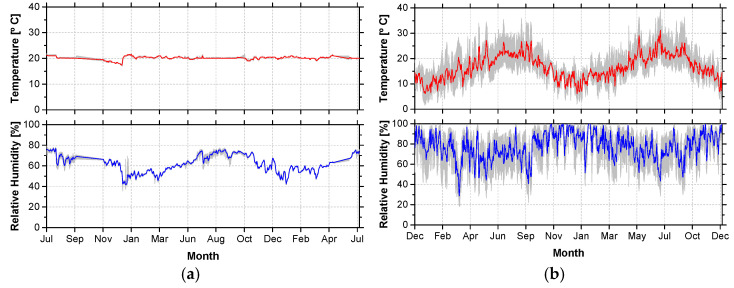
Temperature and relative humidity recorded in environments (**a**) E1 and (**b**) E6.

To control the ambient temperature and relative humidity in each environment, sensors were installed close to the materials. During the recording of the data, some sensors faced technical issues. Due to that, the Portuguese Institute for the Sea and Environment (IPMA) provided the missing information. Table 3 presents the average ambient temperatures and relative humidity, as well as extreme values recorded between the years of 2018 and 2020 for each environment and trimester, while Figure 2 presents two examples of the temperatures and relative humidity recorded.

**Figure 3:**
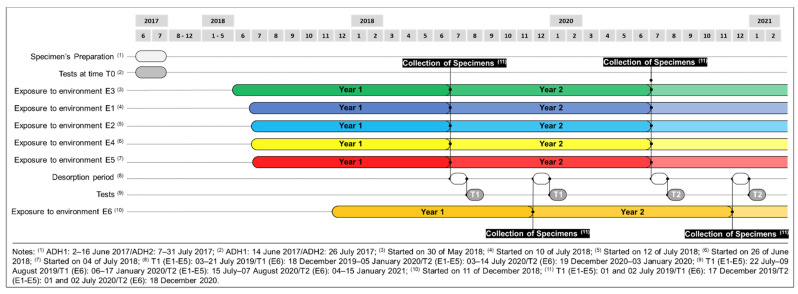
Timeframe of the developed work.

**Figure 4:**
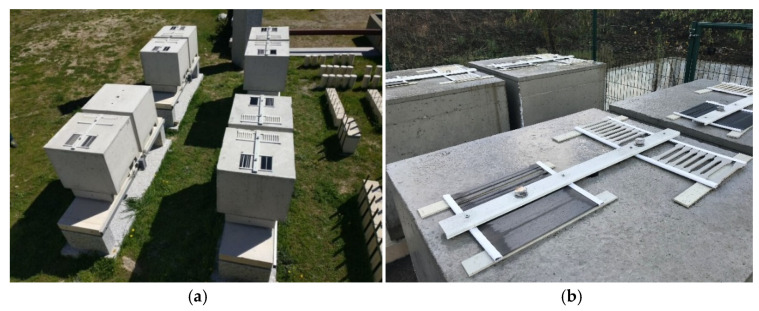
Experimental stations (**a**) E4 and (**b**) E3.

Figure 3 shows a schedule with the main tasks involved in this research. The manufacturing of the epoxy specimens and the preparation of the CFRP laminates took place approximately one year before the beginning of the ageing. During this period, all the specimens were kept in the environment E1. Initial assessment of the material’s mechanical properties was performed at an early age (T0). Thus, epoxy specimens were tested seven days after production (June 2017), while CFRP laminates were tested in July 2017. The installation of the epoxy and CFRP laminate specimens in the experimental stations took place between June 2018 and December 2018 (see Figure 3). A view of two of the experimental stations is provided in Figure 4.

After one (T1) and two (T2) years of exposure, 16 epoxy specimens (eight per type of adhesive) and eight CFRP laminate strips (6 of L10, and 2 of L50) were collected from each environment and tested. The test protocol included a desorption period. Epoxy specimens were sealed onsite and were only exposed to the desorption process one week before testing, with the hygrothermal conditions defined for E1. In the case of CFRP laminates, a three-week desorption period was adopted. During this period, the L50 CFRP strips were cut into the 15 mm wide test specimens. In the case of the specimens immersed in water (E2), no desorption process was adopted and all specimens were kept fully immersed all the time (only being removed immediately before testing). The transportation, desorption process, and test protocol used in both T1 and T2 experimental campaigns was the same, to achieve a direct results comparison and an accurate evaluation of the durability of the materials under each environment.

### 2.3. Methods

#### 2.3.1. Epoxy Adhesives

The unaged (reference) and aged epoxy adhesives were submitted to physical, chemical and mechanical characterization using different methods and techniques, as follows:

The water absorption ability of the unaged epoxy adhesives was assessed by gravimetric measurements up to 10,000 h. Samples of adhesives ADH1 and ADH2 were immersed in water at three different temperatures: 20 °C, 40 °C and 60 °C, according to the ISO 62:2008 [45] methodology. Test specimens, with the same geometry used in DMA experiments (see below), were periodically removed from water and weighed, immediately after being superficially dried, using an analytic balance (AE240 Balance, Mettler Toledo, Greifensee, Switzerland), with 200 g range and readability of 0.1 mg). Subsequently, specimens were returned to the recipients for the continuation of the water absorption process.

**Figure 5:**
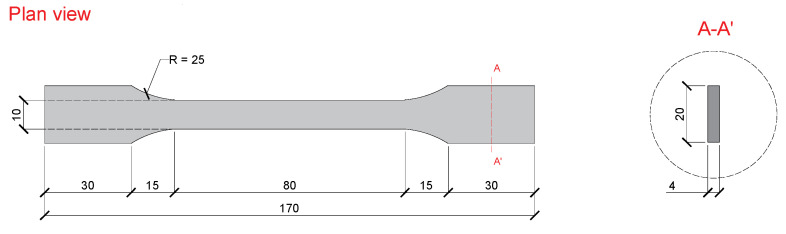
Geometry of the epoxy adhesive specimens used in the tensile tests. All units in (mm).

- The chemical characterization of the unaged epoxy adhesives (cured at 23 °C during 7 days) was performed by Fourier transform infrared spectroscopy (FTIR), according to ASTM E 1252:1998 [46]. Samples were prepared by scraping the surface of tensile specimens (see below); powder obtained were mixed with dry spectroscopy grade potassium bromide and pressed into pellets. FTIR spectra were acquired with a Tensor 27 spectrometer (Bruker, Ettlingencity, Germany) collecting 32 scans at 0.6 cm/s, in the wavenumber range of 4000–450 cm^−1^ with a spectral resolution of 4 cm^−1^. The infrared spectra obtained were compared with spectra available in libraries in order to help the understanding of the position and intensity of the IR absorption bands.
- Dynamic mechanical analysis (DMA) for both unaged (cured at 23 °C, 7 days) and aged epoxy adhesives was carried out in order to evaluate the viscoelastic behaviour of adhesives and determine its glass transition temperature (*T*_g_). The analysis followed ISO 6721-1/5:2019 [47,48]. Prismatic specimens with 4 mm × 10 mm × 60 mm were clamped between the movable and stationary fixtures in a dual cantilever configuration, using a Q800 dynamic mechanical analyser (TA Instruments, New Castle, DE, USA). The tests were conducted in air atmosphere, at a rate of 2 °C/min, from 25 °C to 150 °C. A constant frequency of 1 Hz and a maximum deformation of 15 µm were imposed. Per each adhesive, two replicates were tested. The *T*_g_ was determined using two different methods: (i) as the extrapolated onset of the sigmoidal change in the storage modulus curve (E’), as per in the ASTM E 1640:2018 [49]; and (ii) from the peak of the tan δ curve.
- The tensile tests of both unaged and aged epoxy adhesives were carried out according to the EN ISO 527-2:2012 [50]. The geometry adopted (“type 1A”—as defined in EN ISO 527-2:2012 [50]) is shown in Figure 5. Tests were conducted on a servo-controlled testing machine (model: C11.DE.150KN.100.70.200, INEGI Sentur, Porto, Portugal), equipped with a 10 kN capacity load cell (with a linearity error less than 0.05% F.S.), under displacement control at a rate of 1 mm/min. In each series, composed of five or six specimens, the following strain measure devices were used: (i) a BFLA-5-3-3L strain gauge (gauge length: 5 mm, TML, Tokyo, Japan) glued on the specimen’s geometrical centre and (ii) a clip-on extensometer, with a gauge length of 50 mm (precision of ±1 μm) placed at the central region of constant width of each specimen. The elastic modulus was computed based on the slope between the 0.05% and 0.25% strain, on the stress-strain curve, as defined in the EN ISO 527-1:2019 standard [51].

The fabrication of the epoxy adhesive specimens involved the following steps: (i) manually mixing of the two resin components; (ii) casting of homogenized compound into a Teflon mould; (iii) covering of the top surface of the moulds with acetate sheets and compaction with a steel roller (it should be stressed that all these procedures were executed with the necessary care in order to ensure that all specimens were manufactured with nominal geometry and homogeneity, avoiding the formation of voids and other types of defects); (iv) removal of specimens from the moulds one day after casting, and storage in a climatic chamber under controlled temperature and relative humidity (20 °C and 55% RH).

#### 2.3.2. CFRP Laminates

**Figure 6:**
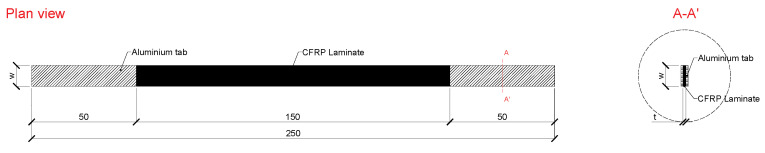
Geometry of the CFRP laminate strip specimens used in the tensile tests. All units in (mm).

The CFRP laminates were submitted to physical and mechanical characterization, using the following methods:

- The water absorption ability of the unaged CFRP laminates with similar procedures to those described for the epoxy adhesive up to 10,000 h, with water at 20 °C.
- The tensile tests of both unaged and aged epoxy adhesives were carried out according to the ISO 527-5:2009 norms [52]. Each specimen was 250 mm long, comprising an initial distance between grips of 150 mm. In order to avoid premature failure due to stress concentration upon the closing of the grips, aluminium tabs of 50 mm were glued to the ends of the CFRP specimens. Figure 6 shows the geometry of the CFRP laminate specimens. The tensile tests were carried out on a servo-controlled testing machine under displacement control with rate of 2 mm/min, equipped with a 200 kN capacity load cell (with a linearity error less than 0.05% F.S.). The following strain measuring devices were used: (i) a clip gauge of 50 mm gauge length (precision of ±1 μm) placed at the central region of constant width of each specimen; and (ii) one strain gauge TML BFLA-5-3-3L, glued on the geometrical centre of one specimen per series. Each series was composed of six specimens. The tensile elastic modulus ($E_{f}$), tensile strength ($f_{f}$), and strain at peak stress ($\varepsilon_{f}$) were determined according to ISO 527-5:2009 [52].

While L10 CFRP strips were tested with their original cross-section geometry (10 mm × 1.4 mm), the L50 tested samples (250 mm × 15 mm × 1.2 mm) were extracted from the unaged/aged original plates (~350 mm × 50 mm × 1.2 mm).

## 3. Results and Discussion

### 3.1. Epoxy Adhesives

#### 3.1.1. Characterization before Exposure (T0)

**Figure 7:**
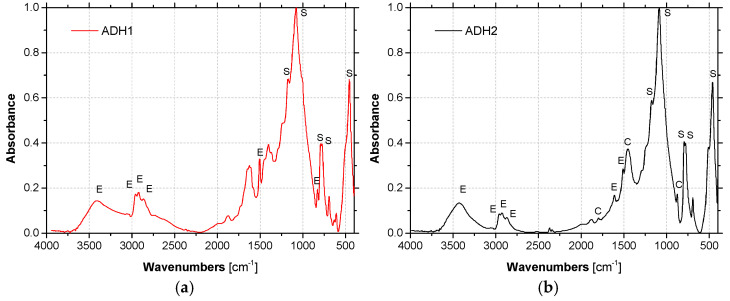
FTIR spectra of unaged adhesives (**a**) ADH1 and (**b**) ADH2 - assignment of IR peaks: E-epoxy, S-silicates and C-carbonates.

Figure 7 shows the results of FTIR spectra for adhesives ADH1 and ADH2. The spectra show consistent peak characteristics of epoxy resin (E), in line with what is stated in the respective technical sheets (both adhesives are filled bicomponent thixotropic adhesives with a bisphenol-A-based resin with an aliphatic amine hardener); it is also possible to identify bands attributed to the presence of silicates (S) in both spectra, and carbonates (C) in ADH2 adhesive. Energy Dispersive X-ray Spectroscopy (EDX) analysis showed that the mineral filler of ADH1 adhesive is quartz [53], and ADH2 adhesive contains, as mineral fillers, calcite and quartz [54].

**Figure 8:**
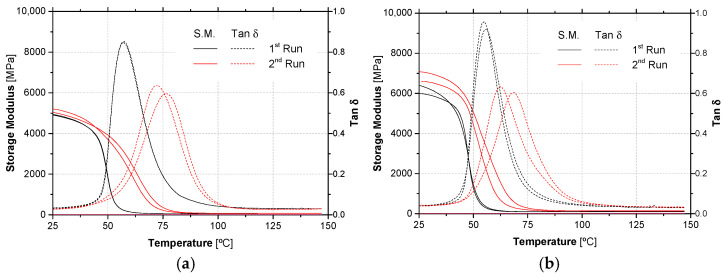
DMA experimental curves of unaged adhesives (**a**) ADH1 and (**b**) ADH2—in two consecutive temperature runs.

Figure 8 presents DMA experimental curves (storage modulus and tan δ versus temperature, two test specimens) for both adhesives ADH1 and ADH2, resulting from two consecutive temperature scans of material (cured at 23 °C for 7 days). Storage modulus curves present the typical sigmoidal shape of polymeric materials: after a glassy plateau, the storage modulus drops sharply and becomes practically null. The corresponding change in the experimental curve of tan δ is a peak.

The first run experimental curve of storage modulus curves started to exhibit a reduction at around 46.2 °C for ADH1 adhesive and 44.3 °C for ADH2 adhesive. On the other hand, tan δ curves exhibited a peak near 57.0 °C for ADH1 adhesive and 55.3 °C for ADH2 adhesive. A study carried out also by DMA with the same adhesives [55], but cured during only 3 days at 21 °C, led to slightly lower *T*_g_ values, but of the same order of magnitude. The corresponding technical data sheet declared 52 °C for ADH2 adhesives, but those value was determined in samples cured at higher temperatures and measured by a distinct method (DSC instead DMA).

The second run experimental curves show an increment in *T*_g_ values for both adhesives: 30% for ADH1 adhesive and 18% for ADH2 adhesive (considering the tan δ values), showing an important post-cure, particularly for ADH1 adhesive.

**Figure 9:**
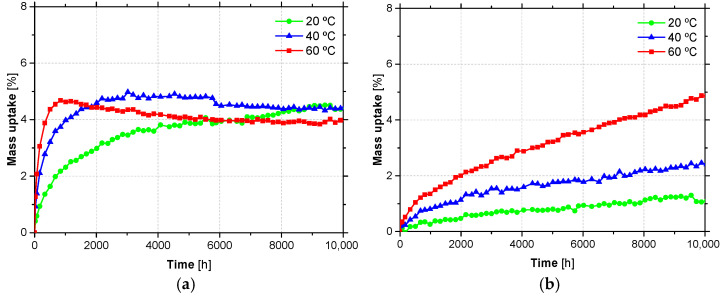
Water absorption of adhesives (**a**) ADH1 and (**b**) ADH2 at 20 °C, 40 °C and 60 °C.

Figure 9 depicted the mass uptake of both adhesives, during water immersion at the three distinct temperatures, namely 20 °C, 40 °C and 60 °C.

As can be seen, the water absorption behaviour of the two adhesives is significantly distinct: while ADH1 adhesive shows a significant absorption rate during the early stages and reaches a steady state equilibrium (fully saturated state) after 5000 h, ADH2 adhesive presents a continuous increase of absorption, particularly for temperatures of 40 °C and 60 °C. As expected, by increasing the temperature, the water absorption increased. However, for immersion at 60 °C, ADH1 adhesive shows a mass loss, probably due to leaching of mineral filler by water. The different water absorption evidenced by the two adhesives, particularly in the initial phase, reflects distinct morphologies of its structure that are strongly affected by the content and nature of the mineral fillers. ADH1 adhesive appears to have a higher free volume to be occupied by water. For this reason, a very rapid initial increase is observed, which tends to settle after 5000 h and, as expected, higher water absorption rates are observed as the temperature increases. A study performed with ADH2 adhesive [56], where water immersion up to 36 months in similar temperatures (23 °C, 37.8 °C and 60 °C) were done, shows an analogous behaviour during the first 10,000 h.

In Section 3.1.2 presents the tensile properties of the epoxy adhesives (ADH1 and ADH2) obtained from the mechanical characterization (tested at T0), in terms of modulus of elasticity (*E*_a_), tensile strength (*f*_a,ult_) and corresponding strain (*ε*_a,ult_). These results show that ADH2 adhesive presents higher mechanical properties, when compared to ADH1 adhesive. The obtained values are lower than the nominal values of the technical data sheet, which is not surprising because it is known that the mechanical properties of this type of adhesives can be affected by several factors occurring during the preparation of test specimens, including curing conditions. The results obtained are in line with those of other studies performed with the same adhesives [57].

#### 3.1.2. Characterization after Exposure

**Figure 10:**
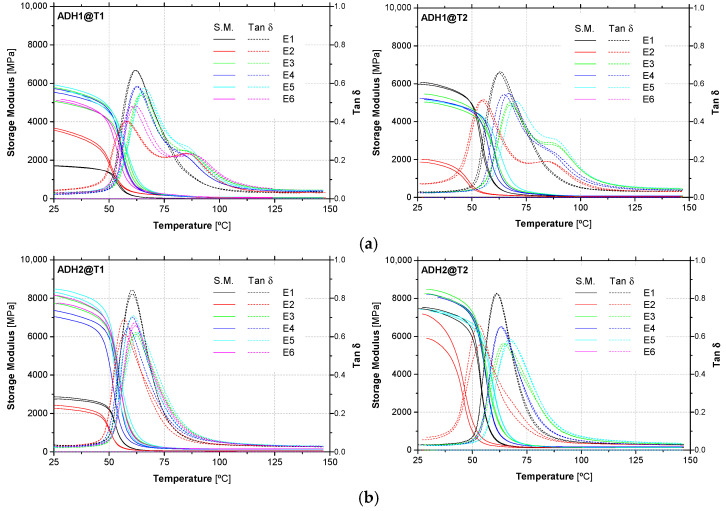
DMA experimental curves of adhesives (**a**) ADH1 and (**b**) ADH2, after one year (T1) and two (T2) years of exposure to the environments studied (E1 to E6).

As detailed in Section 2.3, for each adhesive (ADH1 and ADH2), environment (E1 to E6) and exposure period (T1 and T2), DMA tests were carried out in two replicates. Figure 10 presents the DMA experimental curves obtained.

For ADH1 adhesive, with respect to the peak point, an asymmetry on the right side of the tan δ curves is observed in all types of environments (except for environment E1). This phenomenon is particularly relevant for E2 (water immersion), where a second peak can be observed. This phenomenon (splitting of the tan δ peak curve) has been reported in other investigations (e.g., [56]) about the hygrothermal ageing of polymeric adhesives and is attributed to heterogeneous plasticization. In contrast with the responses observed for ADH1 adhesive, the experimental DMA curves of ADH2 adhesive do not show any asymmetries, related to the presence of water inside the material.

**Figure 11:**
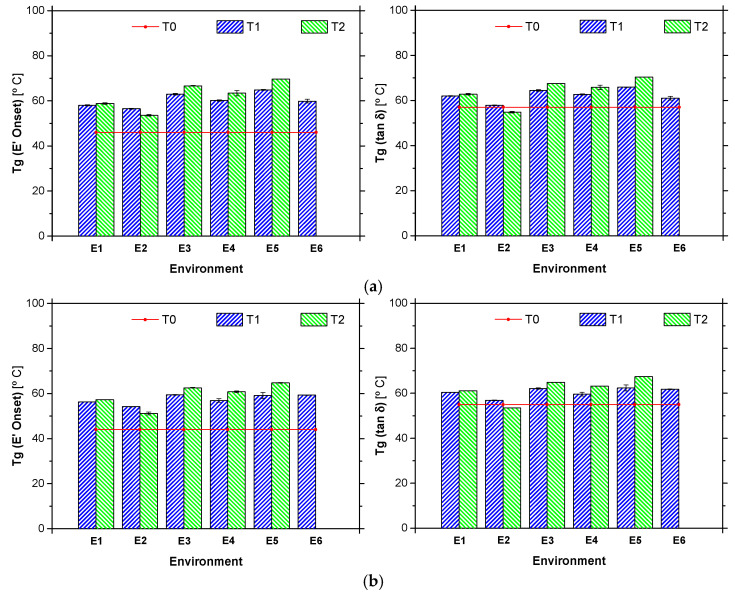
Glass transition temperature of adhesives (**a**) ADH1 and (**b**) ADH2, after one year (T1) and two (T2) years of exposure to the environments studied (E1 to E6), including the reference (cured at 23 °C, 7 days).

Table 4 presents the average values of *T*_g_ obtained from (i) the onset of the storage modulus, *T*_g_ (E’_onset_), and from (ii) the tan δ *T*_g_ (tan δ), for both adhesives (ADH1 and ADH2) and exposure periods (initial characterization—T0, T1 and T2), while Figure 11 presents the same values in the form of graphs, in addition to the initial characterization, considered as reference.

After two years of environmental exposure, the *T*_g_ values of ADH1 adhesive for all outdoor environments (E3 to E6) are still higher than values obtained after curing under standard conditions for 7 days (T0). The reason for such behaviour may be related to the post-curing experienced by the material. When the first (T1) and second (T2) years of exposure are compared, it can be seen that the *T*_g_ values are still increasing, for all outdoor environments. It should be noted, however, that environment E6, which presented the lowest *T*_g_ values for time T1 (probably due to the higher relative humidity in this environment), was not reported and that may present a different trend. ADH1 adhesive exposed to environment E2 yielded to the lowest values of *T*_g_. These results are consistent with the literature (e.g., [53]), where plasticization of the adhesive occurs due to water uptake yielding to a decrease in the *T*_g_ value. Moreover, these results are also consistent with those obtained in tensile tests (see below), where a strong reduction on its tensile properties were observed for this environment.

Similarly to the ADH1 adhesive, in the case of ADH2 adhesive, higher values of *T*_g_ were also observed for all the outdoor environments when compared with reference (curing under standard conditions during 7 days (T0). Moreover, there was an increase on the *T*_g_ values over the last year (from T1 to T2). Also, for the environment E2, a non-negligible reduction in the *T*_g_ values were observed (a decrease of 10.7% for *T*_g_ (E’_onset_) and 12.6% for *T*_g_ (tan δ)), becoming the environment with the lowest values reported.

**Figure 12:**
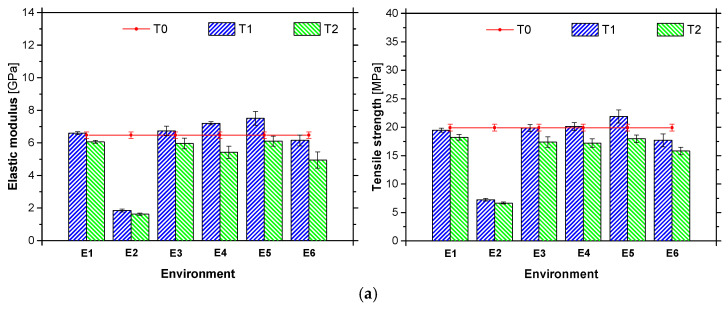

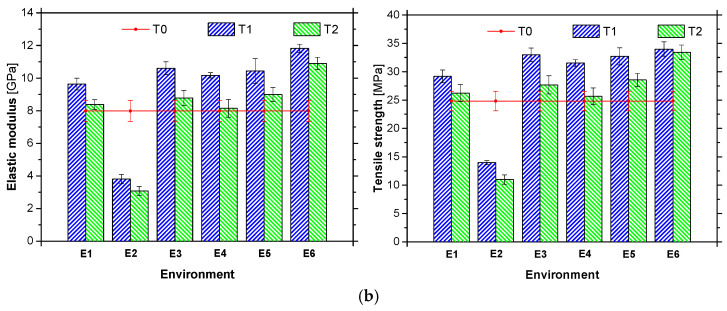
Elastic modulus and tensile strength of adhesives (**a**) ADH1 and (**b**) ADH2, after one (T1) and two (T2) years of exposure to the environment studied (E1 to E6), including the reference (T0).

Table 5 presents the average results obtained from the tensile tests of ADH1 and ADH2 adhesives samples collected in each experimental station, after one (T1) and two (T2) years of environmental exposition. It is also included the values obtained at time T0 (adhesive tested 7 days after casting). Figure 12 shows graphical representation of the tensile strength and the elastic modulus of ADH1 and ADH2 adhesives obtained from the tests performed at T0, T1 and T2.

The mechanical properties of the ADH1 adhesive at time T0 are in agreement with the results obtained in other similar works [53,58,59] and with the ones provided by the supplier [58]. Negligible variations in the values of *f*_ult_ (−2.0%) and *E*_a_ (+1.5%) were observed when the ADH1 adhesive was exposed to the E1 environment for one year (see Figure 12). From T0 to T2, a higher decrease in the mechanical properties was observed on *f*_ult_ (−8.5%) and *E*_a_ (−6.2%), indicating a reduction of the performance of the adhesive even under a controlled environment. The immersion in water (E2 environment) led to the greatest reduction in tensile properties: at T1, specimens exposed to the E2 environment presented a reduction of 62.8% and 72.0% on *f*_ult_ and *E*_a_, respectively, in comparison with the reference E1. This finding was already described in previous studies e.g., [16,20] where the authors showed that the absorption of water by the epoxy can cause plasticization (reduction of the elastic modulus and resistance) and swelling. These effects are more pronounced in the present case since the specimens were tested in a saturated state. From T1 to T2, a reduction on these tensile properties was also observed.

After one year of exposure (T1), ADH1 adhesive presented minor variations in the tensile properties for all outdoor environments (E3 to E6). The one-year exposure to environment E5 led to the highest increase in the mechanical properties of the adhesive (*f*_ult_: +12.4%; and *E*_a_: +13.8%, when compared with E1 specimens tested at T1), probably due to a post-curing phenomenon. As referred in [16,53], the exposure of the epoxy to temperatures higher than the initial curing temperature can lead to a post-curing process, improving the mechanical properties of the adhesive. However, in E6, there was a reduction of the epoxy mechanical properties probably as result of higher humidity registered at this site. During the second year of exposure (between T1 and T2) a general decrease in the tensile properties of ADH1 adhesive was observed in all outdoor environments: again, the E6 environment yielded to the highest reduction on the properties of the adhesive (*f*_ult_: −13.2%; and *E*_a_: −18.4%), probably by the higher humidity, as referred above.

The mechanical properties of ADH2 adhesive at T0 are also in accordance with the values provided by the supplier [59], and with test results from similar works [60]. As shown in Figure 12, there was an increase in the tensile properties of ADH2 adhesive kept on the E1 environment after one year of exposure (*f*_ult:_ +17.7%; and *E*_a_: +17.1%). This increase is an indicator that the curing process of ADH2 adhesive was not concluded after 7 days of curing (test at T0). During the second year of ageing (between T1 and T2), a decrease in the mechanical properties of ADH2 adhesive was observed (*f*_ult_: −10.3%; and *E*_a_: −12.5%) for specimens kept in the reference environment (E1), yet, these results obtained at T2 are higher than those obtained for the unaged (T0) specimens. Similarly to ADH1, the environment E2 led to strong reductions in *f*_ult_ (−52.2% at T1) and *E*_a_ (−60.5% at T1) of ADH2 adhesive, in comparison with the E1 environment, due to the abovementioned reasons. From the T1 to T2, a reduction in *f*_ult_ of −21.3% and *E*_a_ of −19.2% can be observed (for E2). All specimens exposed to the outdoor environments (E3-E6) present an increase in their mechanical properties after one year (T1): the highest increase was observed in E6 (*f*_ult_: +16.5%; and E_a_: +22.6%) and the smallest was observed in E4 (*f*_ult_: +7.9%; and *E*_a_: +6.3%), when compared with E1 specimens at time T1. The effect of relative high temperatures (even in short periods of time), might have caused a post-curing phenomenon. From T1 to T2, a reduction in the performance of the adhesive exposed to the outdoor environments was observed, especially in the E4 environment (*f*_ult_: −18.6%; and *E*_a_: −19.9%). However, the tensile properties of ADH2 adhesive are still higher after the two years of exposure to the outdoor environments, than when tested unaged (T0).

In short, the outdoor environments led to an increase in the mechanical properties of both adhesives (ADH1 and ADH2) during the first year (post-curing phenomenon might be the cause), and a significant decrease after the second year, in contrast with environments E1 and E2 where the degradation agents (moisture and temperature) were controlled, outdoor environments incorporate a synergy between moisture, UV radiation, temperature cycling, and chemical exposure. Although each selected outdoor environment is geographically distant and presents specific ageing conditions (see Section 2.2), the obtained results do not clearly show which one (or degradation agent) has the greatest influence on the adhesive properties. Finally, it should be noted that for both adhesives (ADH1 and ADH2) and exposure times (T1 and T2), immersion in water (E2) led to the greatest degradation of the tensile properties (up to −75%).

### 3.2. CFRP Laminates

**Figure 13:**
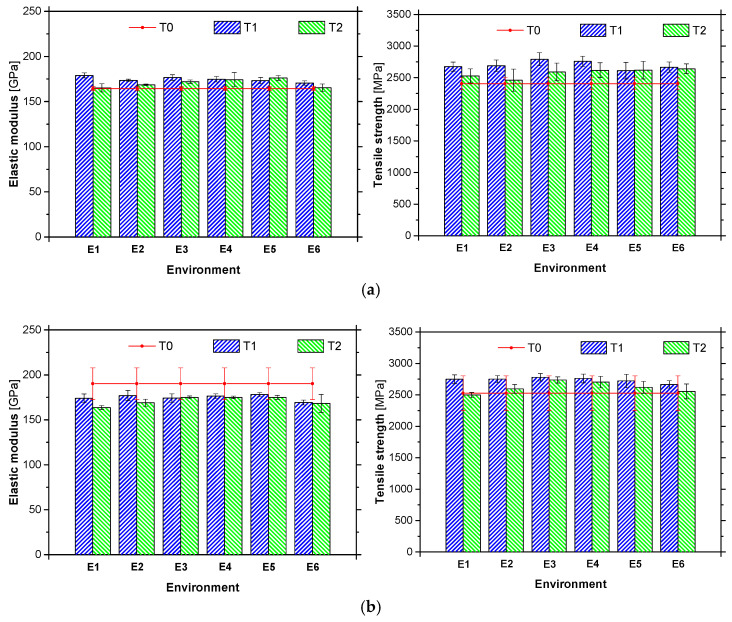
(**a**) Elastic modulus and tensile strength of (**a**) L10 and (**b**) L50 laminates, after one (T1) and two (T2) years of exposure to the environment studied (E1 to E6), including the reference (T0).

CFRP laminates were fully immersed in water at 20 °C for a period of 10,000 h. Test specimens were weighed, on a weekly basis, using a high precision balance (Kern PFB 600-2, company, Balingen, Germany, with 600 g range and readability of 0.01 g), but no mass variation was detected. Considering that the average weigh of a L10 and L50 specimen was 5.52 g and 23.24 g, respectively, the measuring errors are equal to 0.18% for L10 strip and 0.04% for L50 strip. Therefore, it can be stated that the studied CFRP strips present great resistance to the water ingress. Table 6 presents the mean values of the tensile strength (*f*_fu_), elastic modulus (*E*_f_) and strain at peak stress (*ε*_fu_) obtained for the L10 and L50 laminates, after one and two years of environmental exposure. This table also includes the values before ageing (T0), i.e., the reference values. Similarly, Figure 13 shows the mean values of the tensile strength and elastic modulus obtained at T1 and T2 experimental campaigns, alongside with the test results obtained at T0.

The values obtained for *f*_fu_ and *E*_f_ at the time T0 are in agreement with the ones claimed by the supplier. Regardless of the exposure period, in general the type of environment had a minor influence on the mechanical performance of the CFRP laminates (for the same period of exposure, the difference between the maximum and minimum value observed in the different environments is equal to 6.0%). However, when the period of exposure is considered, a tendency of decreasing mechanical properties with the increase of exposure time is observed (an average value of 3.6% was obtained)—a maximum variation of 9.3% was found for the environment E2 between T1 and T2. This variation (from T1 to T2) seems to be higher in the case of strength than in the case of elastic modulus. When T0 is considered in the analysis, in general, for T1 and T2 higher values of the mechanical properties are observed, probably due to a post-curing phase. Finally, regardless the type of environment, the CFRP laminates have presented a low reduction of their mechanical properties.

## 4. Accelerated Ageing versus Natural Ageing

In order to compare the accelerated ageing tests with natural ageing, two databases of accelerated ageing tests (one for epoxy adhesives and another for CFRP laminates) were developed by collecting data from the literature. Then, this data was compared with the results obtained from specimens under natural ageing, developed in the present work.

The database of results of accelerated ageing tests of epoxy adhesive presents the following characteristics:

- More than 105 series (each series composed of three to six specimens) were considered from 17 research works [20,53,56,61,62,63,64,65,66,67,68,69,70,71,72,73,74,75];
- In terms of types of exposure conditions, water immersion, thermal cycles, wet-dry cycles and freeze-thaw cycles were considered;
- Regardless the type of exposure condition, results obtained from tests under temperatures higher than *T*_g_—20 °C were disregarded;
- Water immersion included tap water, demineralised water and water with chlorides;
- Tensile (ISO 527-2, ASTM D638) or flexural (ISO 178, ASTM D790-92) test protocols were adopted for the characterization of the adhesives;
- Periods of exposure up to 21,600 h were found.

**Figure 14:**
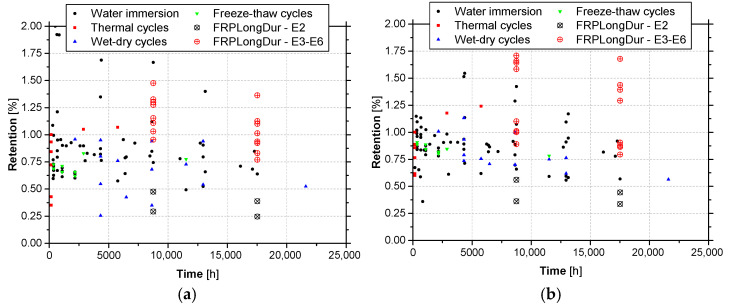
Retention values of (**a**) elastic modulus and (**b**) tensile strength of epoxy adhesives under accelerated ageing tests protocols versus natural ageing (E3 to E6).

Figure 14 depicts the evolution of retention with the time of results in terms the elastic modulus and tensile strength, for epoxy adhesives tested under accelerated ageing protocols. The retention was defined as the ratio between the elastic modulus (or the tensile strength) after ageing and the reference elastic modulus (or the tensile strength), i.e., before ageing. These graphs also include the results of the accelerated ageing tests carried out in the scope of the present work (environment E2) and the results of natural ageing (environments E3 to E6).

Despite the dispersion of results, from these graphs it becomes clear that for similar periods of exposure, accelerated ageing tests yield lower values of the retention parameter, when compared with natural ageing. When retention values lower than 1.0 are considered, average retentions of the tensile strength equal to 0.72 (with a coefficient of variation, CoV = 27%) and 0.88 (CoV = 8.1%) are obtained, respectively, for accelerated ageing and natural ageing (E3 to E6); in the case of elastic modulus, these values are equal to 0.72 (CoV = 27%) and 0.88 (CoV = 4.4%).

When the results of environment E2 are compared with the remaining accelerated ageing tests, in general lower values of retention are obtained in the former tests. The principal reason relies on the fact that the specimens of environment E2 were tests in a wet state, just after being removed from the water recipients, without being submitted to any drying process.

**Figure 15:**
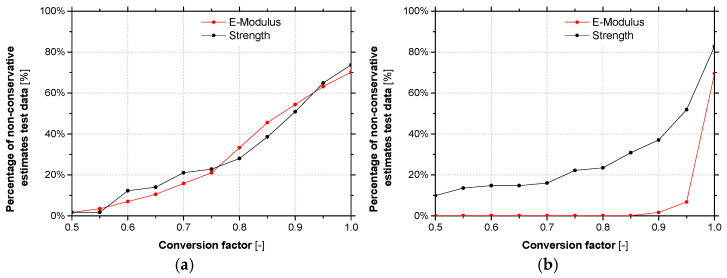
Percentage of non-conservatively estimated data points as function of the conversion factor for (**a**) epoxy adhesives and (**b**) CFRP laminates.

Guidelines, such as ACI 440.2R-17 [5] or CNR-DT 200 R1/2013 [4] use durability conversion factors to account for the detrimental effects caused by the different types of exposure conditions. Based on the data collected from the accelerated ageing tests, non-conservatively estimated data values as a function of the conversion factor were determined and its graphical representation is provided in Figure 15a. Based on these results, for ensuring 10% of the non-conservative estimates, a conversion factor of 0.55 should be adopted.

Regarding to the database of results of accelerated ageing tests of CFRP laminates, the following characteristics can be drawn:

- More than 63 (elastic modulus) and 76 (tensile strength) series—each series composed of three to six specimens—were considered from 14 research works [19,66,75,76,77,78,79,80,81,82,83,84,85,86];
- In terms of types of exposure conditions, water immersion, thermal cycles, wet-dry cycles and freeze-thaw cycles were considered;
- Regardless the type of exposure condition, results obtained from tests under temperatures higher than *T*_g_—20 °C were disregarded;
- Water immersion included tap water, demineralised water and water with chlorides;
- Tensile (ISO 527-5, ASTM D3039/D3039M) or flexural (ISO 14125/ ASTM D7264) test protocols were adopted for the characterization of the CFRP laminates;
- Periods of exposure up to 20160 h were found.

**Figure 16:**
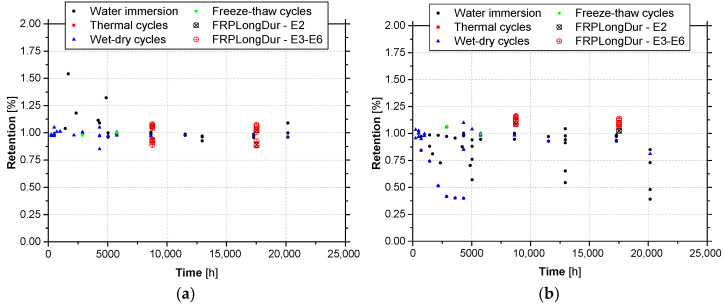
Retention values of (**a**) elastic modulus and (**b**) tensile strength of CFRP laminates under accelerated ageing tests protocols versus natural ageing (E3 to E6).

Figure 16 depicts the evolution of retention with the time of results in terms the elastic modulus and tensile strength, for CFRP laminates tested under accelerated ageing protocols. These graphs also include the results of the accelerated ageing tests carried out in the scope of the present work (environment E2) and the results of natural ageing (environments E3 to E6).

Again, despite the dispersion of results, from these graphs it becomes clear that for similar periods of exposure, in general accelerated ageing tests yield similar or lower values of the retention parameter, when compared with natural ageing. When retention values lower than 1.0 are considered, average retentions of the tensile strength equal to 0.74 (CoV = 42%) are obtained for accelerated ageing, while for natural ageing (E3 to E6) values higher than 1.0 are always obtained; in the case of elastic modulus, these values are equal to 0.97 (CoV = 2.8%) and 0.91 (CoV = 7.9%), respectively. The fact that retention values of elastic modulus for accelerated ageing higher than natural ageing ones are observed may be related to the results obtained in series L50 (see Section 3.2). Moreover, according to these results, the environmental actions have more impact on the strength than on the elastic modulus (see Figure 15b and Figure 16).

## 5. Conclusions

This work addressed the durability of two structural epoxy adhesives (ADH1 and ADH2) and two CFRP laminates (L10 and L50) typically used in strengthening of existing reinforced concrete structures under natural ageing conditions. Four (natural) outdoor environments inducing ageing were adopted, mainly by carbonation (E3), freeze-thaw attack (E4), elevated temperatures (E5), and airborne chlorides from seawater (E6). Furthermore, a control (reference) environment (E1) and an environment involving water immersion of the materials under controlled temperature (E2) were also included in this investigation. The characterization involved the assessment to the physical, chemical and mechanical properties along the time, namely at an early stage (T0) and one (T1) and two (T2) years after exposure to the ageing conditions. Comparisons between the natural ageing tests developed in the scope of the present work and accelerated ageing tests existing in the literature were also performed.

Thus, from the studies carried out with the adhesives ADH1 and ADH2, the following main conclusions can be drawn:

- Results of FTIR spectra of unaged (T0) material have shown typical characteristics of epoxy resins (both adhesives are filled bicomponent thixotropic adhesives with a bisphenol-A-based resin and an aliphatic amine hardener);
- Water absorption tests up to 10,000 h of unaged (T0) material have revealed two completely different behaviors: (i) ADH1 adhesive shows a significant absorption rate at early stages and reaches a steady state equilibrium (fully saturated state) after 5000 h, regardless of the temperature (~5% of uptake), while ADH2 adhesive presented a continuous increase of absorption (at 10,000 h, values of uptake of ~1%, 2.5% and 5% were registered for temperatures of 20 °C, 40 °C and 60 °C, respectively);
- Values of glass transition temperature (based on the onset of sigmoidal change of storage modulus curve from DMA tests), *T*_g_, of unaged (T0) material of 46.2 °C and 44.3 °C were obtained for ADH1 and ADH2, respectively; by performing a post-curing (2nd scan), values of 60.1 °C and 52.3 °C were obtained. Ageing up to two (T2) years, yielded to an increase in the *T*_g_ of the adhesives, regardless of the environment;
- From the tensile tests, values of elastic modulus of 6.5 GPa and 8.0 GPa, and tensile strength of 19.9 MPa and 24.8 MPa, were obtained for the unaged (T0) ADH1 and ADH2, respectively. After one year of exposure (T1), adhesives ADH1 and ADH2 have shown negligible and significant (up to +48%) variations on the tensile properties, respectively, for all the environments (except E2). When compared with T1, for exposure time T2 both adhesives faced a decrease in the tensile properties (up to 25%) for all the environments (except E2). For both adhesives (ADH1 and ADH2) and exposure times (T1 and T2) environment E2 yielded to significant decrease in the tensile properties (up to −75%);
- Despite the dispersion of results, for similar periods of exposure, accelerated ageing tests yield lower values of the retention tensile properties, when compared with natural ageing.

From the studies carried out with the CFRP laminates L10 and L50, the following main conclusions can be highlighted:

- Water absorption tests up to 10,000 h of unaged (T0) material have revealed negligible water uptake values;
- From the tensile tests, values of elastic modulus of 164 GPa and 190 GPa, and tensile strength of 2405 MPa and 2527 MPa, were obtained for the unaged (T0) L10 and L50, respectively. After two years of exposure, CFRP laminates L10 and L50 have shown almost negligible variations of their tensile properties;
- Despite the dispersion of results, for similar periods of exposure, accelerated ageing tests yield similar or lower values of the retention tensile properties, when compared with natural ageing.

Despite the relevant outputs provided by this work, particularly in terms of durability of epoxy adhesives and CFRP laminates under natural ageing conditions, attempts to establish relationships between accelerated and natural ageing test conditions were unsuccessful as they could not to be derived. It may be possible to establish these relationships if longer periods of exposure to natural ageing are adopted. This should be pursued in future research works.

## Figures and Tables

**Table 1:** Adhesives properties.

Property	ADH1 Adhesive		ADH2 Adhesive	
	Value	Test Method	Value	Test Method
Density, at 23 °C [g/cm^3^]	1.7–1.8	*n*/a	1.65	*n*/a
Compression properties Strength [MPa] Modulus [GPa]	>70 *n*/a	EN 12190 [34] --	75; 90 ^(1)^ 9.6 ^(2)^	EN 196 [35] ASTM D 695-15 [36]
Flexural properties Modulus [GPa]	>7.1	EN ISO 178 [37]	*n*/a	--
Tensile properties Strength [MPa] Modulus [GPa]	*n*/a *n*/a	-- --	26; 29 ^(1)^ 11.2 ^(2)^	EN ISO 527-3 [38] EN ISO 527-3 [38]
Shear properties Strength [MPa]	>26	EN 12615 [39]	18 ^(4)^	EN ISO 4624 [40]
Bond-strength by pull-off, on concrete [MPa]	3 ^(3)^	EN 13892-8 [41]	>4 ^(4)^	EN 1542 [42]
*T*_g_ [°C]	*n*/a	--	52 ^(5)^	EN 12614 [43]

Notes: ^(1)^ 7 days at +10 °C; +35 °C; ^(2)^ at 23 °C; ^(3)^ 3 days at 20 °C; ^(4)^ 7 days at +23 °C; ^(5)^ 30 days at 30 °C.

**Table 2:** Environments considered in the present study.

Environment	Location	Description
E1	University of Minho, Guimarães (41°27′11.5″ N 8°17′26.8″ W)	AE; Specimens under controlled hygrothermal conditions, 20 °C and 50% RH (reference environment)
E2	University of Minho, Guimarães (41°27′08.1″ N 8°17′33.8″ W)	AE; Specimens under controlled hygrothermal conditions, 20 °C and 100% RH (immersed in tap water)
E3	National Laboratory of Civil Engineering, Lisbon (38°45′41.7″ N 9°08′30.6″ W)	RE; Mild subtropical Mediterranean climate with short and mild winters and warm to hot summers; high levels of CO_2_
E4	Lagoa Comprida Dam (EDP), Seia (40°21′55.8″ N 7°38′52.0″ W)	RE; Mild subtropical Mediterranean climate with low temperatures and snow during the wintertime and warm to hot summers
E5	Factory of S&P Clever and Reinforcement, Elvas (38°53′33.5″ N 7°08′46.0″ W)	RE; Hot summer Mediterranean climate with high temperatures and drought specially during the summer
E6	Port of Viana do Castelo (APDL), V. do Castelo (41°40′57.0″ N 8°49′28.3″ W)	RE; Mild subtropical Mediterranean climate with short and mild winters and warm to hot summers; high levels of chlorides concentration and RH

**Table 3:** Values of the temperature and relative humidity registered between the years 2018 and 2020 for the different environments studied.

Environment		Year 2018		Year 2019				Year 2020			
		July-September	October-December	January-March	April-June	July-September	October-December	January-March	April-June	July-September	October-December
E1 ^(a)^	Temp. [°C]	20.5 20.0–22.0	18.6 17.5–20.0	20.5 17.0–22.0	20.1 19.5–21.0	20.0 20.0–21.5	20.1 19.0–21.5	20.2 19.0–21.0	20.2 19.0–21.5	--	--
	RH [%]	69.9 55.0–79.5	62.7 53.5–67.5	50.7 35.5–69.5	60.6 49.0–74.0	71.5 56.5–77.5	63.8 51.5–75.0	54.3 41.5–60.5	62.5 48.5–75.5	--	--
E2 ^(b)^	Temp. [°C]	24.3 24.1–24.5	21.4 19.7–25.0	20.6 18.6–21.4	21.2 19.8–23.9	21.6 20.9–22.4	19.8 17.6–21.6	20.0 ^(3)^ -	20.0 ^(3)^ -	--	--
	RH [%]	100.0 -	100.0 -	100.0 -	100.0 -	100.0 -	100.0 -	100.0 -	100.0 -	--	--
E3 ^(c)^	Temp. [°C]	22.2 ^(1)^ 12.7–46.2	15.3 6.2–34.3	17.1 3.3–25.7	17.9 6.8–35.3	22.0 14.5–39.7	15.7 6.8–31.0	13.6 4.3–27.0	14.9 6.6–23.5	--	--
	RH [%]	66.2 ^(1)^ 14.0–100.0	78.5 11.0–100.0	71.0 12.0–100.0	67.1 13.0–100.0	66.8 20.0–100.0	81.6 19.0–100.0	78.3 22.0–100.0	80.5 34.0–100.0	--	--
E4 ^(2) (d)^	Temp. [°C]	18.0 7.4–32.4	7.8 −2.8–22.2	5.8 −4.7–18.5	10.2 −3.1–26.7	17.1 4.8–29.6	7.7 −2.3–24.6	6.1 −4.6–19.2	11.5 −3.6–27.0	--	--
	RH [%]	60.3 4.0–100.0	78.8 16.0–100.0	63.9 7.0–100.0	71.4 5.0–100.0	59.4 8.0–99.0	80.5 7.0–100.0	75.3 4.0–100.0	79.6 14.0–100.0	--	--
E5 ^(2) (e)^	Temp. [°C]	26.1 12.6–44.6	13.2 1.1–33.0	10.9 −1.9–26.2	19.3 4.7–38.0	25.0 11.7–39.9	14.5 3.1–34.7	11.6 0.3–25.3	19.2 2.1–39.0	--	--
	RH [%]	49.1 9.0–95.0	79.8 11.0–100.0	69.7 14.0–100.0	54.6 10.0–100	48.5 11.0–97.0	75.8 1.04–100.0	78.2 28.0–100.0	66.5 11.0–100.0	--	--
E6 ^(a)^	Temp. [°C]	--	--	12.1 1.5–28.5	17.9 5.0–34.5	21.8 12.0–36.0	13.7 3.5.0–28.0	12.6 2.0–25.0	19.0 5.5–25.0	22.5 11.0–39.5	14.2 4.0–26
	RH [%]	--	--	76.1 18.0–100.0	69.0 26.5–99.0	71.0 22.0–99.0	88.1 45.0–100.0	82.8 38.5–100.0	75.8 33.5–100.0	70.2 28.5–99.5	86.7 42.0–100.0

Notes: For each environment, the mean value (first line) and the extreme minimum and maximum values (second line) are provided; ^(1)^ Also considered 26–30 June 2018; ^(2)^ Values obtained from IPMA (the IPMA’s location station is 9 km apart E3 and 560 m apart E5); ^(3)^ Values obtained from the sensor installed on the experimental station; The following sensors were used to record the data: ^(a)^ EL-USB-2 EasyLog USB Data Logger (Akron, OH, USA) with a range of −35 to +80 °C for temperature and 0 to 100% for humidity (RH); ^(b)^ Carel PT100 Thermocouple (Padova, Italy) with a range of −50 to +250 °C for temperature; ^(c)^ Thies Clima 1.1005.54.000 (Göttingen, Germany) with a range of −30 to +70 °C for temperature and 0 to 100% for humidity (RH); ^(d)^ MicroStep-MIS PT100 (Bratislava, Slovakia) with a range of −50 to +70 °C for temperature and 0 to 100% for humidity (RH); ^(e)^ Vaisala HUMICAP® HMP155 (Vantaa, Finland) with a range of −80 to +60 °C for temperature and 0 to 100% for humidity (RH).

**Table 4:** Glass transition temperature of adhesives ADH1 and ADH2, after one year (T1) and two (T2) years of exposure to the environments studied (E1 to E6), including the reference (T0).

Environment	*T*_g_ (E’_onset_) [°C] (CoV [%])			*T*_g_ (tan δ) [°C] (CoV [%])		
	T0	T1	T2	T0	T1	T2
**ADH1**						
E1	46.2 (0.3)	50.4 (1.1)	50.5 (0.6)	57.0 (0.2)	62.0 (0.2)	62.8 (0.6)
E2		46.6 (0.1)	43.2 (1.0)		57.9 (0.3)	54.9 (0.6)
E3		51.8 (0.2)	54.1 (0.5)		64.5 (0.6)	67.6 (0.1)
E4		51.5 (0.3)	54.9 (2.0)		62.7 (0.5)	65.8 (1.5)
E5		51.2 (1.1)	56.2 (-)		65.9 (0.2)	70.4 (-)
E6		49.9 (1.6)	*n*/a		61.1 (1.4)	*n*/a
**ADH2**						
E1	44.3 (1.0)	49.9 (0.0)	50.5 (0.4)	55.3 (0.8)	60.5 (0.1)	61.2 (0.2)
E2		46.8 (0.0)	42.3 (0.8)		56.9 (0.3)	53.5 (1.2)
E3		50.1 (0.2)	51.0 (0.4)		62.1 (0.5)	64.9 (0.9)
E4		49.3 (2.7)	52.0 (0.5)		59.6 (1.5)	63.2 (0.2)
E5		48.9 (0.7)	53.9 (0.5)		62.4 (2.2)	67.4 (0.3)
E6		50.3 (0.3)	*n*/a		61.8 (0.2)	*n*/a

**Table 5:** Tensile properties of adhesives ADH1 and ADH2, after one (T1) and two (T2) years of exposure to the environment studied (E1 to E6), including the reference (T0).

Environment	*f*_ult_ [MPa] (CoV [%])			*E*_a_ [GPa] (CoV [%])			ε_ult_ [%] (CoV [%])		
	T0	T1	T2	T0	T1	T2	T0	T1	T2
**ADH1**									
E1	19.9 (3.0)	19.5 (1.8)	18.2 (2.8)	6.5 (3.0)	6.6 (1.3)	6.1 (1.4)	0.4 (6.2)	0.4 (13.0)	0.3 (11.6)
E2		7.2 (3.1)	6.7 (2.7)		1.9 (5.2)	1.6 (4.0)		1.1 (21.3)	1.1 (11.9)
E3		19.9 (3.1)	17.4 (5.3)		6.7 (4.4)	6.0 (5.4)		0.3 (11.1)	0.3 (19.1)
E4		20.1 (3.4)	17.2 (4.3)		7.2 (1.4)	5.4 (6.9)		0.3 (11.3)	0.3 (12.8)
E5		21.9 (5.2)	18 (3.6)		7.5 (5.7)	6.1 (5.0)		0.3 (11.2)	0.3 (13.1)
E6		17.7 (6.4)	15.8 (4.3)		6.2 (5.4)	5.0 (10.0)		0.3 (4.3)	0.3 (12.9)
**ADH2**									
E1	24.8 (7.0)	29.2 (3.8)	26.2 (5.7)	8.0 (8.2)	9.6 (3.7)	8.4 (3.6)	0.4 (20.0)	0.3 (18.5)	0.4 (18.2)
E2		13.9 (2.5)	11.0 (7.6)		3.8 (7.4)	3.1 (8.8)		0.3 (10.8)	0.3 (11.7)
E3		33.0 (3.6)	27.7 (5.8)		10.6 (3.7)	8.8 (5.3)		0.4 (14.7)	0.3 (8.7)
E4		31.5 (1.8)	25.7 (5.7)		10.2 (1.6)	8.1 (6.6)		0.4 (9.1)	0.3 (15.8)
E5		32.7 (4.6)	28.6 (4)		10.4 (7.2)	9.0 (4.7)		0.4 (10.3)	0.3 (14.4)
E6		34.0 (3.8)	33.4 (3.8)		11.8 (2.1)	10.9 (3.4)		0.3 (11.3)	0.3 (3.5)

**Table 6:** Tensile properties of L10 and L50 laminates, after one (T1) and two (T2) years of exposure to the environment studied (E1 to E6), including the reference (T0).

Environment	*f*_fu_ [MPa] (CoV [%])			*E*_f_ [GPa] (CoV [%])			*ε*_ult_ [×10^−3^] (CoV [%])		
	T0	T1	T2	T0	T1	T2	T0	T1	T2
**Laminate L10**									
E1	2405 (3.8)	2674 (2.7)	2528 (4.4)	164 (1.2)	179 (1.6)	165 (2.7)	14.6 (3.8)	14.9 (3.2)	15.3 (6.1)
E2		2688 (3.4)	2460 (7.1)		174 (0.7)	168 (0.5)		15.5 (2.9)	16.0 (12.5)
E3		2792 (3.7)	2590 (5.4)		177 (1.8)	172 (1.1)		15.8 (3.8)	15.1 (5.1)
E4		2758 (2.9)	2617 (4.5)		175 (1.8)	174 (4.5)		15.8 (2.3)	15.0 (4.5)
E5		2611 (5.0)	2619 (5.3)		173 (2.0)	176 (1.5)		15.1 (4.6)	14.9 (5.2)
E6		2667 (3.0)	2640 (2.9)		171 (1.4)	165 (4.2)		15.6 (2.9)	16.0 (1.9)
**Laminate L50**									
E1	2527 (10.8)	2748 (2.6)	2497 (1.7)	190 (9.3%)	174 (2.8)	164 (1.3)	13.3 (13.6)	15.8 (3.6)	15.3 (1.9)
E2		2750 (2.0)	2594 (2.8)		177 (3.2)	169 (2.3)		15.6 (3.9)	15.5 (3.0)
E3		2778 (2.1)	2735 (1.8)		174 (2.7)	175 (0.9)		16.0 (3.6)	15.6 (1.2)
E4		2760 (2.5)	2703 (3.4)		176 (1.5)	175 (0.8)		15.7 (2.8)	15.4 (3.3)
E5		2720 (3.9)	2618 (3.6)		178 (1.2)	175 (1.3)		15.3 (4.0)	14.9 (2.6)
E6		2665 (2.2)	2554 (4.6)		169 (1.6)	168 (6.0)		15.7 (2.6)	15.2 (6.1)

## Data Availability

Not applicable
